# Improved dynamic distortion correction for fMRI using single‐echo EPI and a readout‐reversed first image (REFILL)

**DOI:** 10.1002/hbm.26440

**Published:** 2023-08-07

**Authors:** Simon Daniel Robinson, Beata Bachrata, Korbinian Eckstein, Saskia Bollmann, Steffen Bollmann, Shota Hodono, Martijn Cloos, Monique Tourell, Jin Jin, Kieran O'Brien, David C. Reutens, Siegfried Trattnig, Christian Enzinger, Markus Barth

**Affiliations:** ^1^ Centre of Advanced Imaging University of Queensland Brisbane Australia; ^2^ Department of Neurology Medical University of Graz Graz Austria; ^3^ High Field MR Centre, Department of Biomedical Imaging and Image‐Guided Therapy Medical University of Vienna Vienna Austria; ^4^ Karl Landsteiner Institute for Clinical Molecular MR in Musculoskeletal Imaging Vienna Austria; ^5^ Department of Medical Engineering Carinthia University of Applied Sciences Klagenfurt Austria; ^6^ School of Information Technology and Electrical Engineering The University of Queensland Brisbane Australia; ^7^ ARC Training Centre for Innovation in Biomedical Imaging Technology (CIBIT) The University of Queensland Brisbane Australia; ^8^ Siemens Healthcare Pty Ltd. Brisbane Australia

**Keywords:** dynamic distortion correction, fMRI, susceptibility, ultra‐high field

## Abstract

The boundaries between tissues with different magnetic susceptibilities generate inhomogeneities in the main magnetic field which change over time due to motion, respiration and system instabilities. The dynamically changing field can be measured from the phase of the fMRI data and corrected. However, methods for doing so need multi‐echo data, time‐consuming reference scans and/or involve error‐prone processing steps, such as phase unwrapping, which are difficult to implement robustly on the MRI host. The improved dynamic distortion correction method we propose is based on the phase of the single‐echo EPI data acquired for fMRI, phase offsets calculated from a triple‐echo, bipolar reference scan of circa 3–10 s duration using a method which avoids the need for phase unwrapping and an additional correction derived from one EPI volume in which the readout direction is reversed. This Reverse‐Encoded First Image and Low resoLution reference scan (REFILL) approach is shown to accurately measure *B*_0_ as it changes due to shim, motion and respiration, even with large dynamic changes to the field at 7 T, where it led to a > 20% increase in time‐series signal to noise ratio compared to data corrected with the classic static approach. fMRI results from REFILL‐corrected data were free of stimulus‐correlated distortion artefacts seen when data were corrected with static field mapping. The method is insensitive to shim changes and eddy current differences between the reference scan and the fMRI time series, and employs calculation steps that are simple and robust, allowing most data processing to be performed in real time on the scanner image reconstruction computer. These improvements make it feasible to routinely perform dynamic distortion correction in fMRI.

## Graphical abstract

We present an improved method for dynamic distortion correction of single‐echo fMRI data called REFILL which requires two reference scans of just 3 s (GRE) and one EPI TR. REFILL accurately corrects distortions due to dynamic changes to the field, increasing time‐series SNR in data in which there is substantial motion.

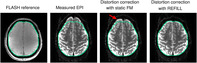

## 1 INTRODUCTION

The interfaces between tissues of the head with different magnetic susceptibilities generate an inhomogeneous magnetic field, *B*_0_ (Purcell & Morin, 2013). The resulting variation in the Lamour frequency throughout the object leads to signal loss and distortion in images. Distortion is particularly prominent in the phase‐encode direction in Echo Planar Imaging (Mansfield, 1977) due to the low bandwidth. In fMRI, distortions cause coregistration errors (Gartus et al., 2007), a reduction in BOLD sensitivity in group studies (Cusack et al., 2003) and the mislocalization of eloquent cortex in pre‐surgical planning (Lima Cardoso et al., 2018). In the classic, static FLASH (fast, low‐angle shot)‐based field mapping approach (Frahm et al., 1986), the *B*_0_ distribution throughout the object is estimated from a multi‐echo gradient‐echo acquisition via the phase evolution (Jezzard & Balaban, 1995). Another widely used method is to estimate the distortion field from a pair of images in which the phase‐encoding direction is reversed (a.k.a. gradient reversal, blip up/down, TOPUP) (Andersson & Skare, 2002). Other static approaches are based on mapping the Point Spread Function (Zaitsev et al., 2006; Zeng & Constable, 2002), the centre of which is shifted in the presence of field offsets, and diverse artificial intelligence techniques (Hu et al., 2020; Liao, 2018). Such static distortion correction methods do not, however, capture temporal changes in *B*_0_ over the course of the fMRI session due to drift (Foerster et al., 2005), physiological fluctuations (van Gelderen et al., 2007), and motion (Dymerska et al., 2018; Hagberg et al., 2012; Liu et al., 2018).

Ultra‐high field MRI systems are becoming more prevalent in use in fMRI due to higher SNR and sensitivity to susceptibility effects (Beisteiner et al., 2011; Triantafyllou et al., 2005; van der Zwaag et al., 2009); features which can be taken advantage of clinically following recent regulatory approval of 7 T systems from two vendors. As well as providing higher BOLD contrast, though, the increased sensitivity to susceptibility effects exacerbates artefacts related to inhomogeneity and temporal changes in *B*_0_, including distortion. To address this, dynamically acquired information relating to *B*_0_ has been used to correct the detrimental effects of macroscopic field variations in magnetic resonance spectroscopy (Bogner et al., 2014), magnetic resonance spectroscopic imaging (MRSI) (Boer et al., 2012), chemical exchange saturation transfer (Poblador Rodriguez et al., 2019) and diffusion‐weighted imaging (Avram et al., 2014). Dynamic field mapping can also inform dynamic shimming (Stockmann & Wald, 2018) with higher order and matrix shims (Aghaeifar et al., 2018; Juchem et al., 2010; Kim et al., 2017) and integrated radio‐frequency (RF) receive and shim coil arrays (Han et al., 2013; Stockmann et al., 2015). Recent years have seen a trend to towards higher resolution fMRI (de Martino et al., 2018), in which precise localization of activation is of central interest, and to ultra‐high field (Huber et al., 2017; Uğurbil, 2018). Dynamic changes to the field are larger in this regime, refocussing efforts to dynamically measure *B*_0_ during the acquisition in order to accurately correct image distortion.

Some of the dynamic methods described above are not well suited to fMRI. The need for rapid sampling in fMRI renders the volumetric navigators used in anatomical (Tisdall et al., 2012) and MRSI sequences (Bogner et al., 2013) impractical. Multi‐echo EPI allows a field map to be calculated from each time point (Visser et al., 2012), but single‐shot, multi‐echo EPI is limited in the achievable spatial resolution, particularly given the shorter *T*_2_* at ultra‐high field. Low‐order field changes can be detected with ^19^F‐based NMR probes (Wilm et al., 2015) but this does not capture high spatial frequency components and the need for additional hardware makes this, currently, a niche option. The field can be modelled using free induction decay navigators of just a few milliseconds (Wallace et al., 2021; Wallace, Polimeni, et al., 2020), but further work is needed to integrate such an approach into sequences for routine application. Ideally, it would be possible to calculate accurate dynamic field maps from the phase of single‐echo fMRI data without additional hardware or complex modelling of the field.

The necessity, in most *B*_0_‐mapping methods, to acquire the phase at multiple echo times arises from the need to eliminate the time‐invariant, non‐*B*_0_‐related contributions to the phase, known as the ‘initial phase’ or ‘phase offset’. The phase offset comprises receive coil sensitivities, which are different for each coil, and contributions from gradient delays, eddy currents and transmit RF phase, all of which affect all coil signals identically (Robinson et al., 2016). Single‐echo dynamic field mapping approaches (Hahn et al., 2009; Lamberton et al., 2007; Marques & Bowtell, 2005; Ooi et al., 2013) have been proposed predicated on the idea that, in contrast to the *B*_0_ field itself, phase offsets are quite stable over time, particularly at low and intermediate field strengths (≤3 T). The validity of this assumption was subsequently confirmed in a study by Dymerska et al. (2018), showing that, even with shorter RF wavelength at 7 T, changes to phase offsets arising even from large shifts in head position are nearly two orders of magnitude smaller than the associated changes in *B*_0_. As such, accurate field maps can be generated from single‐echo EPI if phase offsets can be determined and removed. That study demonstrated a proof‐of‐principle implementation of single‐echo dynamic distortion correction (DDC), but the method was subject to a number of disadvantages which pose a barrier to its routine application. First, it required two gradient‐echo prescans with reversed gradient polarity to separate phase offsets from the residual phase gradients in the readout direction that can arise from gradient delays, eddy currents and imperfectly centred signals in k‐space. Those prescans had the same resolution as the EPI so that, despite parallel imaging acceleration, the acquisition time was nearly 90 s. The prior method entailed phase unwrapping in two steps: in the calculation of phase offsets from the FLASH reference data and the generation of time‐series field maps from channel‐combined phase images. The first unwrapping step was problematic because it had to be carried out on low SNR separate‐channel data. This made it error‐prone due to the reduced coverage of each channel phase image, and a procedure which precluded a fast and robust integration into the image reconstruction. Finally, no correction was made for the phase gradient in the readout direction in EPI which can occur due to eddy currents, timing errors and phase corrections applied in reconstruction.

The aim of this study was to improve single‐echo DDC to make it practical to apply the method routinely in fMRI. The improved approach requires just one very fast reference scan for the calculation of coil sensitivities with a modified method which requires no phase unwrapping. All non‐*B*_0_‐related contributions to the phase in the EPI are removed using information gleaned from the readout‐reversed volume and the approach is insensitive to shim differences between the FLASH reference scan and the EPI. Together, these improvements make it feasible to apply DDC routinely in fMRI with no additional hardware and minimal additional measurement time.

## 2 THEORY

**FIGURE 1:**
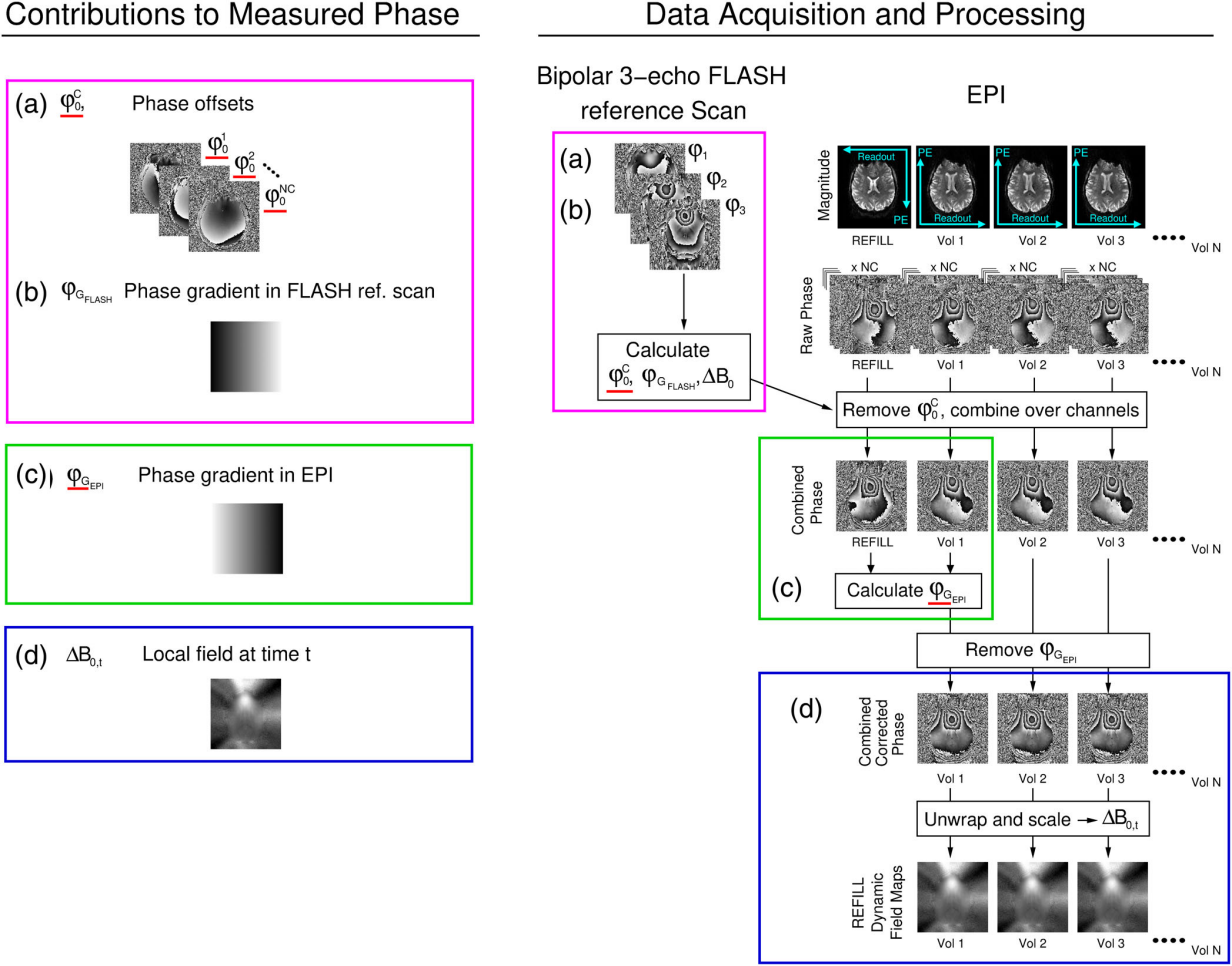
Contributions to the phase in FLASH and EPI data and how they are determined in the REFILL dynamic field mapping approach. Left: The phase measured at time *t* comprises (a) coil‐dependent phase offsets (b/c) sequence‐dependent gradients in the readout direction and (d) the local field at the time of measurement. Right: Data acquisition and calculations. A low resolution, bipolar 3‐echo FLASH reference scan (right panel) is acquired prior to the fMRI time‐series data (labelled ‘EPI’). The reference scan data is used to calculate (a) phase offsets and (b) and readout‐related phase gradient for the FLASH reference scan. In the EPI time series, an initial (REFILL) volume in which the readout (and optionally the phase‐encode direction) is reversed (see cyan axes of the REFILL volume and Vol 1 in the EPI magnitude images) is used to calculate (c) the readout‐related phase gradient for EPI. The parameters which are required for the calculation of (d) REFILL field maps—$\varphi_{0}^{c}$ and $\varphi_{G\_\mathrm{EPI}}$—are underlined in red. Note that the magnitude of $\varphi_{G\_\text{FLASH}}$ was increased for this illustration to aid visualisation.

In this section, we describe how dynamic field maps can be calculated from the phase of the single‐echo EPI time series used for fMRI given knowledge of the phase offsets affecting each coil, $\varphi_{0}^{c}$, and the phase gradient in the readout direction in EPI, $\varphi_{G\_\mathrm{EPI}}$. $\varphi_{0}^{c}$ is calculated from a fast multi‐echo FLASH reference scan and $\varphi_{G\_\mathrm{EPI}}$ from a single EPI volume in which the readout direction is reversed (see Figure 1). We begin by considering the constituents of the phase measured in FLASH and EPI and their relation to local variations in the main magnetic field, $∆B_{0}$.

The phase of the complex‐valued MR signal, θ, is inherently wrapped into a range of 2*π* radians given by $\left.\left(\varphi_{L},\varphi_{L}+2\pi \right.\right]$, where $\varphi_{L}$ is the lower limit, which is usually chosen to be −π. In the following description, θ is used for wrapped phase and φfor phase which is not wrapped. The two are related by

(1) $$\theta =\left(\varphi -\varphi_{L}\right)\;\mathrm{mod}2\pi +\varphi_{L}$$

and

(2) φ=θ+n2π,

where *n* is the number of wraps which have occurred.

In a gradient‐echo sequence with bipolar readouts (i.e. in which the signal is sampled under both positive and negative readout gradient lobes), the phase of the *j*th echo acquired with a surface coil *c* of an RF array with *C* coils, is given by

(3) $$\theta_{j}^{c}=\left(T_{\mathrm{Ej}}\cdot \gamma \Delta B_{0}+\varphi_{0}^{c}+{\left(-1\right)}^{j}\phi_{G_{\text{FLASH}}}+\pi \right)\;\mathrm{mod}\;2\pi -\pi ,$$

where $T_{\mathrm{Ej}}$ is the echo time of the *j*th echo, γ is the gyromagnetic ratio, $\varphi_{0}^{c}$ is the phase offset of coil *c*, −π is adopted for $\varphi_{L}$ and $\Delta B_{0}$ is the field offset in Tesla (and all other quantities are also in SI units). We use $\varphi_{0}^{c}$ to refer to the phase at time zero which is identical in acquisitions under odd and even echoes. The term $\phi_{G\_\text{FLASH}}$, which also contributes to the initial phase, describes the linear phase gradient in the readout direction which arises from eddy currents, timing errors, other sources of echo shift in the acquisition and reconstruction. It depends on acquisition parameters and corrections applied in image reconstruction and has opposite polarity for even and odd readouts (Huber et al., 2017; Tisdall et al., 2012). It is necessary to calculate $\varphi_{G\_\text{FLASH}}$ in order to calculate $\theta_{0}^{c}.$

Given a bipolar multi‐echo sequence with at least 3 echoes, where ${\mathrm{TE}}_{j}=j\times ∆\mathrm{TE}$ and ∆TE is the echo spacing, the wrapped phase offset, $\theta_{0}^{c}$ (which can substitute for $\varphi_{0}^{c}$ in Equation (1) due to the mod2π operation) can be calculated using the ASPIRE method (Eckstein et al., 2018):

(4) $$\theta_{0}^{c}=2\theta_{2}^{c}-\theta_{1}^{c}+3\phi_{G\_\text{FLASH}},$$

where a wrapped version of $\phi_{G\_\text{FLASH}}$, which we will call $\theta_{G\_\text{FLASH}}$, is given by

(5) $$\theta_{G\_\text{FLASH}}=\frac{\left(2\theta_{2}^{c}-\theta_{1}^{c}-\theta_{3}^{c}+\pi \right)\mathrm{mod}2\pi -\pi }{4}.\;$$

$\theta_{G\_\text{FLASH}}$ is unwrapped by exploiting the linearity of $\phi_{G}$, as described in Eckstein et al. (2018).

Using Equation (1) with complex notation to facilitate summation over coils, a dynamic series of field maps can be calculated from the phase of each EPI time point, $\theta_{t}^{c}$:

(6) $$∆B_{0,t}=\frac{1}{\gamma ∙{\mathrm{TE}}_{\mathrm{EPI}}}\left(∠\sum_{c}{\left(M_{t}^{c}\right)}^{2}∙\exp\left[i∙\left(\theta_{t}^{c}-\theta_{0}^{c}+\varphi_{G\_\mathrm{EPI}}\right)\right]+n2\pi \right),$$

in which $\theta_{0}^{c}$ is calculated from the FLASH reference scan (Equation (5)), the squared magnitude $M_{t}^{c}$ provides weighting by an estimate of the coil sensitivities, ∠ denotes the angle of the complex‐valued sum and n is determined using a phase unwrapping algorithm (Robinson et al., 2016). The remaining unknown in Equation (6) is $\varphi_{G\_\mathrm{EPI}}$, the readout phase gradient in EPI. In the proposed REFILL approach, $\varphi_{G\_\mathrm{EPI}}$ is calculated from two EPI volumes with opposite readout polarities, e.g. between an initial reverse‐encoded volume (with the subscript REFILL) and the first regular volume in the time‐series (with the subscript 1):

(7) φG_EPI=12∠∑cM1c∙MREFILLc∙expiθ1c−θREFILLc.

## 3 METHODS

The contributions to the measured phase and data acquisition in the REFILL dynamic field mapping approach are illustrated in Figure 1.

This study was approved by the Ethics Committee of the University of Queensland. All volunteers participated with written informed consent. Data were acquired with a Siemens 7 T Plus scanner (Siemens Healthineers, Erlangen, DE) with a 32‐channel head coil (Nova Medical, Wilmington, MA, USA). Imaging parameters are listed in Table 1 for the following experiments.

**TABLE 1:** Imaging parameters in experiments 1–4.

Experiment #	Protocol #	Sequence	Matrix size (+reconstructed)	# slices	Slice thickness (mm)	TE (ms)	TR (ms) + (FatSAT) + (NVols)	GRAPPA factor	Bandwidth (Hz)	Readout gradient polarity	TA (s)
1	1a	2D FLASH	128 × 128	5	3	{3:3:36}		2	250	{LR,RL}	14
	1b	2D FLASH	128 × 128	5	3	6		2	{200:100:2000}	{LR,RL}	14
	1c	2D EPI	128 × 128	5	3	{21:3:39}	1000	2	1565	{LR,RL}	4
	1d	2D EPI	128 × 128	5	3	30	1000	2	{1000:200:2000}	{LR,RL}	4
2	2a	2D FLASH	128 × 128	40	3	{2.5:2.5:7.5}	856 (FS)	2	600	Bipolar	53
	2b	2D FLASH	128 × 128	40	3	{2.5:2.5:7.5}	394	2	600	Bipolar	25
	2c	2D FLASH	128 × 128	40	3	{2.5:2.5:7.5}	400	4	600	Bipolar	21
	2d	3D FLASH	128 × 128	40	3	{2.5:2.5:7.5}	10	2 × 2	600	Bipolar	17
	2e	2D FLASH	128 × 128	40	3	{2.5:2.5:7.5}	400	6	600	Bipolar	14
	2f	2D FLASH	96 × 96	20	3	{2.5:2.5:7.5}	193	4	600	Bipolar	5
	2g	2D FLASH	64 × 64	20	3	{2.5:2.5:7.5}	96	4	595	Bipolar	3
	2h	2D EPI	128 × 128	5	3	30	2270	2	1565	{LR,RL}	13
3 and 4	3a, 4a	2D FLASH	128 × 128	40	3	{2.5:2.5:7.5}	386	4	600	Bipolar	14
	3b, 4b	2D FLASH	64 × 64 (128 × 128)	40	3	{2.5:2.5:7.5}	386	4	600	Bipolar	9
	3c, 4c	2D EPI	128 × 128	5	3	22	2270		1565	RL	13
	3d, 4d	2D EPI	128 × 128	5	3	22	2270 (1, 100)		1565	LR	243
	Shim change										
	3e	2D FLASH	128 × 128	40	3	{2.5:2.5:7.5}	386	4	600	Bipolar	14
	3f	2D EPI	128 × 128	5	3	22	2270 (1)		1565	RL	13
	3g	2D EPI	128 × 128	5	3	22	2270 (1)		1565	LR	13

*Note*: LR—readout gradient orientation left → right. RL—readout gradient orientation right → left. TA—acquisition time. Values in curly brackets indicate multiple measurements {lowest value:increment:highest value}. The field of view was 210 mm for all scans. The nominal resolution was 1.6 mm for 128 matrix size, 2.2 mm for 96 matrix size and 3.3 mm for 64 matrix size.

*Experiment 1: Investigation of the magnitude and parameter dependence of phase gradients in the readout direction in gradient‐echo and echo‐planar images*, $\phi_{G\_\text{FLASH}}$ *and* $\phi_{G\_\mathrm{EPI}}$.

TE and RBW are parameters which were hypothesised might affect eddy currents and acquisition timing, and thereby echo centring and readout gradient. Axial single‐echo FLASH and 2D EPI data of a spherical oil phantom were acquired with a range of TEs and receiver bandwidths (RBW). Two scans were acquired for each TE and RBW, with the readout gradient orientation left → right and right → left, that is, with the readout gradient along the same direction but with opposite polarity.

*Experiment 2: Dependence of the accuracy of dynamic distortion field maps on the pre‐scan resolution and acceleration*. Bipolar multi‐echo reference scans were acquired with a range of resolutions, acceleration factors and the use of fatsat in one 51‐year‐old healthy female subject.

*Experiment 3: Comparison of REFILL with FLASH‐based static distortion correction*, *accuracy of REFILL field maps in the context of a change in shim and comparison with TOPUP*.

### 3.1 Comparison of REFILL with FLASH‐based static distortion correction and accuracy in the context of a change in shim

Static and dynamic field mapping was performed in three subjects (two female, average age 39.3 years). A multi‐echo gradient‐recalled echo (ME‐FLASH) scan was acquired for the comparison method, static distortion correction (SDC) (protocol 3a in Table 1). Scans for the dynamic method comprised a fast 2D ME‐FLASH reference scan (protocol 3b), a single EPI volume with first readout line R → L (protocol 3c) followed by a time series of 100 volumes with first readout line L → R (protocol 3d).

The effect of respiration and realistic natural changes in head position on *B*_0_ were assessed in one additional subject (male, age 25 years) for whom field maps (protocol 3a) were acquired on inhalation breath‐hold, exhalation breath‐hold and after a small change in head position, for which the instruction was to rotate the head about the left–right axis, sinking the chin by approximately 5 mm.

The accuracy of dynamic field maps calculated with REFILL (Equation (10)) is dependent on phase offsets $\left(\varphi_{0}^{c}\right)$ and readout gradients for the FLASH $\left(\phi_{G_{\text{FLASH}}}\right)\;$and EPI $\left(\phi_{G_{\mathrm{EPI}}}\right)\;$being unaffected by changes in shim. This was tested in one healthy subject (female, aged 51), where a shim change was imposed to mimic the effects of drift and subject change of position, after which reference ME‐FLASH data were acquired again (to provide a comparison static $∆B_{0\_\mathrm{GE}}$ for the new shim condition – protocol 3e) followed by two volumes of EPI with opposite readout polarity (protocols 3f and 3 g).

### 3.2 Comparison of REFILL with the phase gradient‐reversal method TOPUP

Rather than mapping the local field via the effect this has on the signal phase, a distortion field can be calculated from two images with opposing phase‐encode direction which show equal and opposite distortion, the most commonly used implementation of which is TOPUP (Andersson & Skare, 2002). TOPUP requires the acquisition of a single EPI volume in which the phase‐encode direction is reversed, whereas REFILL requires a volume in which the readout direction is reversed. Both TOPUP and REFILL can, however, use a volume in which both the phase‐encode and readout directions are reversed. The data acquired in Experiment 3 were used to compare the FLASH fieldmap (FLASH‐FM) with the TOPUP‐FM and REFILL‐FM.

*Experiment 4: Accuracy and BOLD sensitivity of REFILL‐corrected time series in the presence of field changes*.

The same 3 subjects as in Experiment 3 performed a task in which they moved both hands from their sides to close to (but not touching) their chin with an approximate period of 15 s (self‐paced) to induce dynamic changes to the field (Wallace, Afacan, et al., 2020). SDC information was acquired with protocol 4a and DDC and the fMRI time series with protocols 4b, 4c and 4d.

### 3.3 Analysis

Data acquired in experiments 1–3 were reconstructed using the standard vendor's reconstruction and separate channel phase and magnitude images were exported. For these experiments, data was processed offline in MATLAB (Mathworks Inc., Natick, MA), including upscaling of $\varphi_{0}^{c}$ where the reference scan resolution was less than that of the EPI. In typical fMRI experiments it is impractical to export single‐channel data for offline processing, as these are typically represented as NS × NC × NT × 2 files, where NS = number of slices, NC = number of coils in the receive array, NT = number of time points and 2 counts phase and magnitude images. Performing coil combination on the image reconstruction computer and using tiled images comprising all slices (‘mosaic’ format) reduces the number of files by a factor of NS × NC, which is typically of the order of 1000, to two files per TR (phase and magnitude). To allow this for functional data (Experiment 4), REFILL DDC was implemented on the scanners' image reconstruction computer in the vendor's image reconstruction environment, ‘ICE’. To allow REFILL to be applied to a range of EPI scans to which the user may not have the source code, the reference scan, single EPI with reversed readout and EPI time series were acquired as separate scans. The steps in the calculation of $\varphi_{G\_\mathrm{EPI}}$ and $∆B_{0,t}$ (equations (6) and (7)) were modified slightly from those in the Theory section so that they could be performed offline, on channel‐combined data, as follows. The bipolar, triple‐echo FLASH reference scan data were used to calculate $\varphi_{0}^{c}$ online (Equation (3)) and save these for reading by a modified EPI image reconstruction, also online, which removed $\varphi_{0}^{c}$ and combined the data over channels:

(8) $$\theta_{t}^{\prime }=∠\sum_{c}{\left(M^{c}\right)}^{2}∙\exp\left[i∙\left(\theta_{t}^{c}-\theta_{0}^{c}\right)\right].$$

These interim phase images were exported offline, and the remaining calculations, which were performed on RF channel‐combined data, were carried out using MATLAB:

(9) φG_EPI=12∠expiθ1′−θREFILL′

and

(10) $$∆B_{0,t}=\frac{1}{\gamma ∙{\mathrm{TE}}_{\mathrm{EPI}}}\left(∠\exp\left[i∙\left(\theta_{t}^{\prime }+\varphi_{G_{\mathrm{EPI}}}\right)\right]+n2\pi \right).$$

*n* was determined and wraps removed using ROMEO (Dymerska et al., 2021), a path‐based phase‐unwrapping algorithm which identifies a reliable ‘seed voxel’ in the image and proceeds, voxel by voxel, on a path defined by the quality of the connection between voxels and determining, from the difference between neighbouring phase values (the absolute value of which should be less than π radians) whether −1, 0 or +1 wraps have occurred, and adding the corresponding number of multiples of 2*π* radians to the voxel under consideration.

Field maps are only accurate within the signal‐generating volume, so need to be masked and interpolated to remove noisy values and generate useful estimates at the edge of the brain. For FLASH‐based static field maps, masks were defined by applying BET (Smith, 2002) to the first echo magnitude image and eroding by one voxel. BET was found to create inaccurate brain outline estimates for EPI magnitude images due to the signal inhomogeneity and distortion. For EPI‐based field maps, a single mask for each run (rather than one per field map) was generated from the first image by thresholding the combined quality image from ROMEO (Dymerska et al., 2021), which is scaled from 0 to 1, at a value of 0.5. For both SDC and DDC, field maps were masked by setting all values outside the mask and values outside the range −600 rad/s < $∆B_{0,t}$ < 2000 rad/s to NaN prior to smoothing with the function ‘smoothn.m’ in MATLAB (Garcia, 2009); an operation which omits NaN values from the smoothing kernel and replaces them with smoothed values.

*Experiment 1: Parameter dependence of phase gradients in the readout direction in gradient‐echo and echo‐planar images*, $\phi_{G\_\text{FLASH}}$ *and* $\phi_{G\_\mathrm{EPI}}$. $\phi_{G\_\text{FLASH}}$ and $\phi_{G\_\mathrm{EPI}}$ were calculated from pairs of measurement with opposing readout gradient polarities, as in Equation (7). Mean values of $\phi_{G\_\text{FLASH}}$ and $\phi_{G\_\mathrm{EPI}}$ were calculated over all readout lines in each measurement.

The hypothesis that the phase gradient in the readout direction, $\phi_{G}$, results predominantly from imperfectly centred k‐space was explored as follows. Separate‐channel phase and magnitude image data were reconstructed using the manufacturer's method and read into memory in MATLAB (Mathworks, Natick, USA). These were converted to k‐space data by applying 2D FFT to the image data for each slice and channel. This complex data was shifted by half the matrix size in the readout and phase‐encode directions and interpolated to a size of 2 × RO+1 in the readout direction, where RO was the original reconstructed matrix size in the readout direction. All k‐space readout lines were summed and the difference between the position of the maximum and the centre of k‐space determined. All readout lines in the full k‐space data were shifted by this difference prior to inverse Fourier transform and resampling to a matrix size of RO in the readout direction to regenerate well‐centred image space data.

*Experiment 2: Dependence of the DDC on the prescan resolution and acceleration*. REFILL *B*_0_ field maps were calculated from the first regular EPI volume using phase offsets derived from each of seven prescans with a range of resolutions and parallel imaging acceleration factors. A reference FLASH field map can be calculated from the Hermitian inner Product of two echoes *α* and *β* with the same readout polarity using (Jezzard & Balaban, 1995; Robinson & Jovicich, 2011)

(11) $$∆B_{0}=\frac{1}{2\pi \gamma ∙∆{\mathrm{TE}}_{\beta -\alpha }}\left(∠\sum_{c}M_{\beta }^{c}∙M_{\alpha }^{c}∙\exp\left[i∙\left(\theta_{\beta }^{c}-\theta_{\alpha }^{c}\right)\right]+n2\pi \right).$$

Echoes 1 (*α*) and 3 (*β*) were used and n was determined (and removed) with ROMEO (Dymerska et al., 2021). The difference between this FLASH field map and the REFILL field maps was calculated inside a brain mask generated as described in the first section of the analysis.

*Experiment 3: Comparison of REFILL with FLASH‐based static distortion correction*, *accuracy of REFILL field maps in the context of a change in shim and comparison with TOPUP*.

Each field map was used to remove distortions in the first regular EPI time point; using linear interpolation for REFILL and FSL's FUGUE for the static FLASH‐based approach. REFILL field maps (which were in the distorted space) were undistorted using linear interpolation with MATLAB's interp1 function prior to comparison with FLASH field maps. Differences between REFILL FMs and FLASH field maps were evaluated in voxels which were inside both the EPI and the FLASH masks.

In the assessment of the effect of changes in head position, field maps were coregistered via the corresponding first echo magnitude images using FSL's FLIRT (Jenkinson et al., 2002) and root‐mean‐squared voxel displacements between the two acquisition assessed with FSL's ‘rmsdiff’.

TOPUP field maps were generated according to the TOPUP guide (https://fsl.fmrib.ox.ac.uk/fsl/fslwiki/topup). Additionally, to visualise the distortion field, a grid of lines traversing the readout direction (*x*) at 5 voxel intervals was generated using MATLAB, distorted using the FLASH field map using linear interpolation. REFILL and TOPUP were applied to remove these distortions.

*Experiment 4: Accuracy and BOLD sensitivity of REFILL‐corrected time series in the presence of field changes*. Time‐series EPI were distortion‐corrected using REFILL DDC and SDC using FSL's fugue. Non‐distortion‐corrected, REFILL‐corrected and SDC‐corrected time series were motion corrected using FSL's mcflirt prior to calculation of the standard deviation over time. In a functional analysis of the same data, MELODOC ICA (Beckmann, 2012) was performed for each subject and used to identify the time course of hand motion, and these used as regressors in a general linear model analysis with FEAT (Smith et al., 2004), which was performed on data which had been coregistered to the MNI152 template using FLIRT (Jenkinson et al., 2002) and smoothed with a Gaussian filter with FWHM = 3 mm.

## 4 RESULTS

*Experiment 1: Investigation of the magnitude and parameter dependence of phase gradients in the readout direction in gradient‐echo and echo‐planar images*, $\phi_{G\_\text{FLASH}}$ *and* $\phi_{G\_\mathrm{EPI}}$.

**FIGURE 2:**
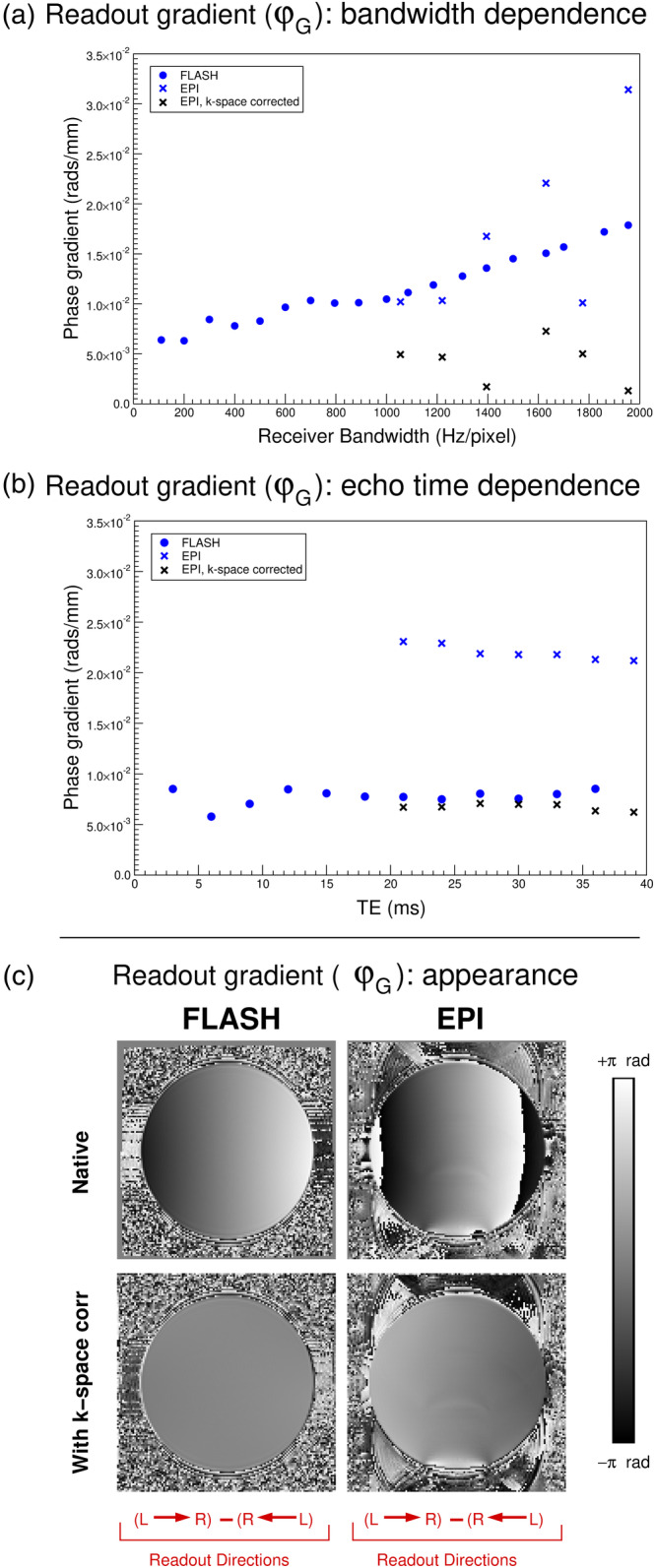
Dependence of the readout gradient, $\varphi_{G}$, on sequence (FLASH, EPI), receiver bandwidth (a) and echo time (b) and appearance of the readout gradient in example FLASH and EPI measurements (c). There was a strong dependence on readout bandwidth (a) and little dependence on echo time (b). For EPI, the readout direction (e.g. L → R) refers to the first acquired k‐space line. Values for k‐space shift‐corrected EPI were smaller for all measurements and are shown in the plots (black crosses). For FLASH, no shift was applied for many points (mainly the smaller values) because only integer shifts were applied and the optimum shift was 0; those values are not shown to avoid crowding the plots. The readout phase gradients $\varphi_{G\_\text{FLASH}}$ and $\varphi_{G\_\mathrm{EPI}},$each calculated from a pair of acquisitions with opposing readout gradients (labelled ‘native’) could be reduced by up to a factor of 10 by shifting the position of the maximum in k‐space (labelled ‘With k‐space corr’ in C; n.b. a constant value of π rad was added to the FLASH ‘With k‐space corr’ image to allow the images to be visualised over the same range). Note that, in the absence of a correction, $\varphi_{G}$ translates to error in the field map of $\varphi_{G}/\left(2\mathrm{\pi TE}\right)$ Hz.

The phase gradient in the readout direction, $\varphi_{G}$, varied with receiver bandwidth and, to a lesser extent, TE (plots in Figure 2). Values ranged from 1 × 10^−3^ rads/mm to 3.2 × 10^−2^ rads/mm; the latter being equivalent to 55 Hz across the 210 mm FOV at 20 ms, a typical TE for EPI at 7 T. Sample images of FLASH and EPI are shown before and after k‐space shift corrections. For the FLASH image (which corresponds to the point in A) with bandwidth = 1860 Hz/pixel), $\varphi_{G\_\text{FLASH}}$ was 0.0171 rad/voxel prior to correction, and 0.00177 rad/voxels afterwards, a 10‐fold reduction. For the EPI example (which corresponds to the point in B) with TE = 21 ms), $\varphi_{G\_\mathrm{EPI}}$ was 0.0379 rad/voxel prior to correction and 0.00671 rad/voxel afterwards, a reduction by a factor of 5.6. These findings confirm that $\varphi_{G}$ (i) is different for FLASH and EPI acquisitions, (ii) is a function of acquisition parameters, (iii) is founded in an imperfectly centred k‐space and (iv) is sufficiently large to need correction in an accurate DDC method.

*Experiment 2: Dependence of the accuracy of dynamic distortion field maps on the prescan resolution and acceleration*.

The accuracy of REFILL field maps was unaffected by the resolution or acceleration factor of the reference scans, the use of fat saturation or whether the scan was 2D or 3D (Supporting Information Figure S1). Looking at the extremes of resolution and acquisition times, 2D reference scans with large voxels (3.2 mm × 3.2 mm × 12 mm) and parallel imaging acceleration factor 4 (acquisition time of 3 s) yielded field maps which agreed equally well with the reference field maps as those generated with unaccelerated reference scans of the same resolution as the EPI (acquisition time of 53 s).

**FIGURE 3:**
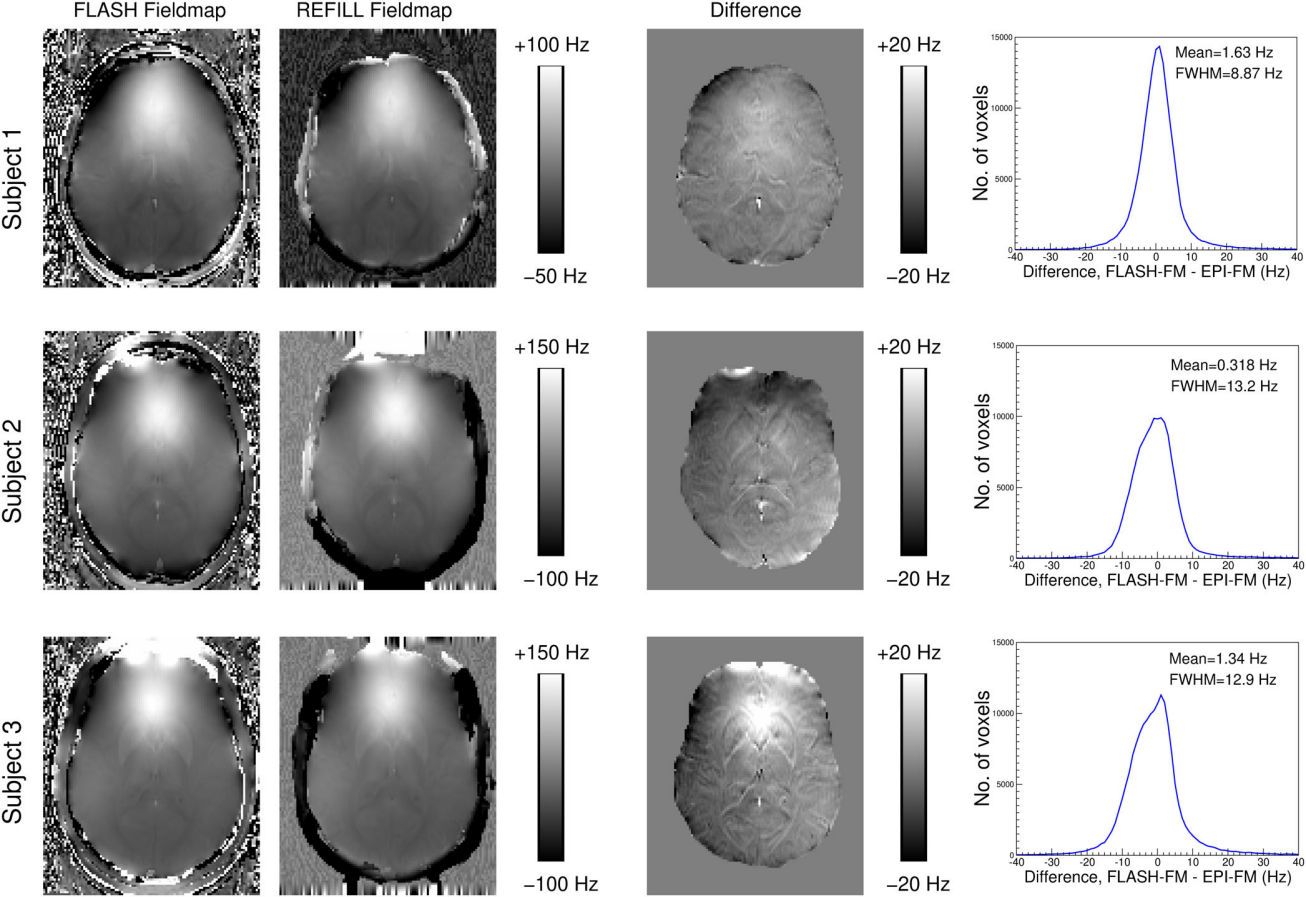
Assessment of the accuracy of EPI‐based field maps in three subjects. The first EPI‐based REFILL field map from each subjects' resting‐state measurement was compared with FLASH field maps acquired with the same shim. Differences, assessed within a brain mask, showed good agreement between the two, with most voxels in the brain agreeing to within a few Hz (see histograms). The images of difference point to higher actual resolution for the FLASH field maps and regional variations indicative of small field changes between the two acquisitions.

In the absence of deliberate shim change, there was good agreement between EPI‐based field maps which were generated with the proposed REFILL method and unwarped to bring them to undistorted space and FLASH‐based field maps. The differences between the two are shown in Figure 3, for subjects S1–S3. Histograms of the difference (in the same figure), show that agreement was generally within a few Hz, with the FWHM being 11.7 ± 2.4 Hz on average over subjects. The images reveal the higher actual resolution in the FLASH‐based field maps (which were acquired with the same nominal resolution), due to PSF blurring in EPI (Schmitt et al., 1998) and some regional variations which are different for each subject and are suggestive of slight changes in the field due to respiration and motion. These discrepencies were investigated further. The difference between field maps acquired at inhalation and exhalation was extremely small—with a FWHM of the histogram of the difference being less than 1 Hz (Figure S2). A small head movement (a root mean squared displacement of voxels of 2.43 mm), on the other hand, led to a FWHM difference of 4 Hz, a substantial fraction of the observed difference between FLASH‐based and REFILL field maps.

*Experiment 3: Comparison of REFILL with FLASH‐based static distortion correction*, *accuracy of REFILL field maps in the context of a change in shim and comparison with TOPUP*.

**FIGURE 4:**
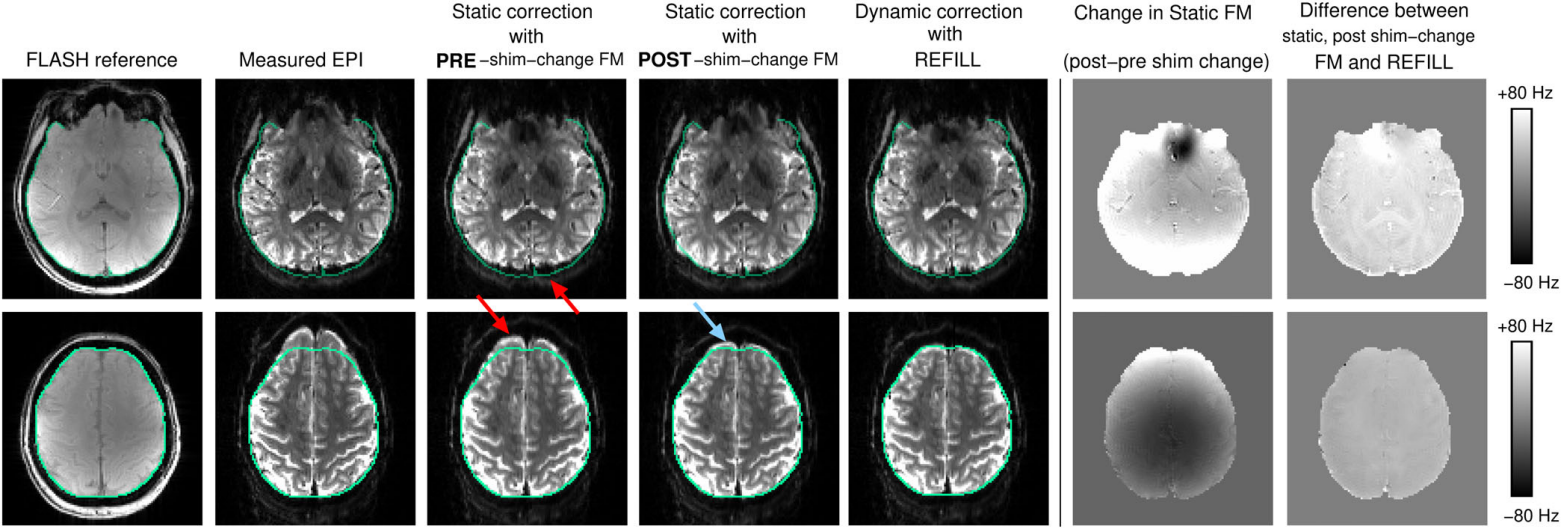
Effect of shim change on the accuracy of a conventional (static) FLASH‐based field map and the dynamic EPI‐based REFILL field map. An imposed shim change, mimicking the effect of motion or physiology in fMRI, led to a change in the field of up to ±80 Hz (second column from the right) and additional distortion in occipital and frontal regions (second column). A correction with the pre‐shim‐change field map was, as is to be expected, incomplete (third column, at red arrows). The dynamic REFILL field map for this volume was accurate and gave a complete correction (fourth column)—see outline transferred from the distortion‐free FLASH reference image.

Figure 4 shows that REFILL field maps were also accurate when a shim change of up to ±80 Hz was imposed between the reference scans and the EPI (mimicking motion or respiration‐related changes in *B*_0_), whereas the conventional static FLASH‐based field maps acquired prior to the shim change did not, of course, capture that change, and show an imperfect distortion correction (see green contour and red arrows in the third column). The post‐shim‐change static FM led to a correction which was generally good but incomplete in the upper slice (bottom row, fourth column, blue arrow), possibly pointing to there having been an additional change in the field (e.g. due to subject motion) between the fieldmap and the EPI. Discrepancy between the post‐shim‐change FLASH‐based field map and REFILL field map was small (see difference map and scale), demonstrating that REFILL captures dynamic changes in the field even when the multi‐echo reference data, which are used in the REFILL fieldmap calculation ($\theta_{0}^{c}$ in Equation (9)), were acquired prior to the change in shim.

**FIGURE 5:**
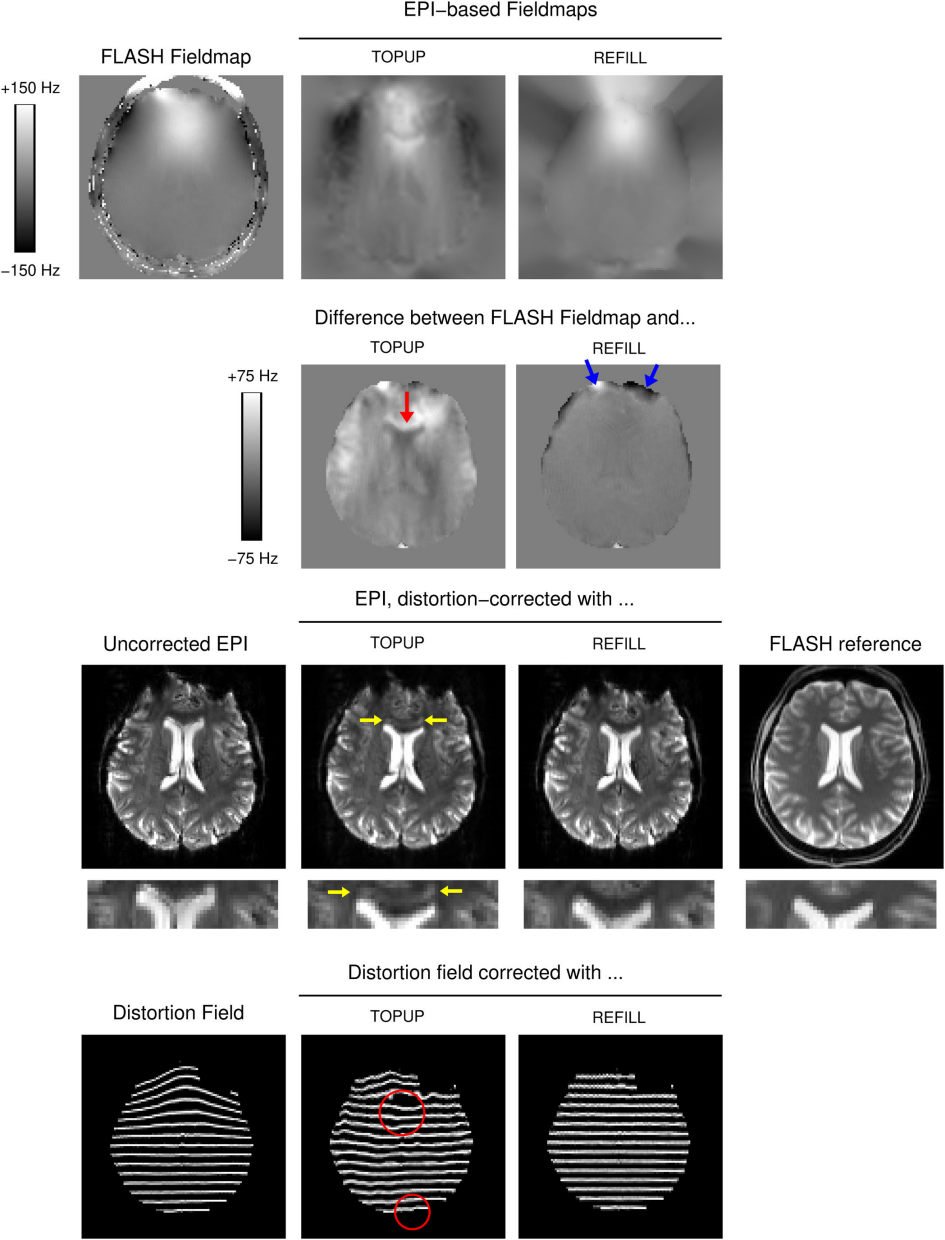
Comparison of REFILL and TOPUP field maps with a reference FLASH field map. The TOPUP field map shows streaking and structure around the ventricles which is not present in the FLASH field map, particularly apparent in the difference image in the second row (at yellow arrow). This generated a shadowing of the ventricles when applied to distortion‐correct the EPI (yellow arrows in zoomed image in fourth row) and residual errors in correcting the distortion field (bottom and red circles). The REFILL field map agreed with the FLASH field map to within a few Hz other than at the edge of the image (blue arrows). The EPI was well corrected (note the position of the ventricles compared to the FLASH reference in the zoomed image). The distortion field can also be seen to be well corrected, with residual minor discrepancies attributable primarily to interpolation.

REFILL field maps agreed with reference FLASH field maps to a much better degree than TOPUP field maps (Figure 5). With TOPUP, the disparity in field estimates was up to 50 Hz over substantial regions of the brain, with field map errors of high spatial frequency leading to a shadowing of the ventricles in the corrected image (yellow arrow). Other than these, the TOPUP image appears well corrected (see the position of the ventricles in the zoomed image), but residual distortions are clearly apparent in the ‘distortion field’ (bottom row and red circles). The REFILL field map agreed with the FLASH reference field map to within a few Hz over most of the image with significant deviations only at low signal regions close to the edge of the image (blue arrows), which did not affect the quality of the correction (see e.g. position of the ventricles in the zoomed image).

**FIGURE 6:**
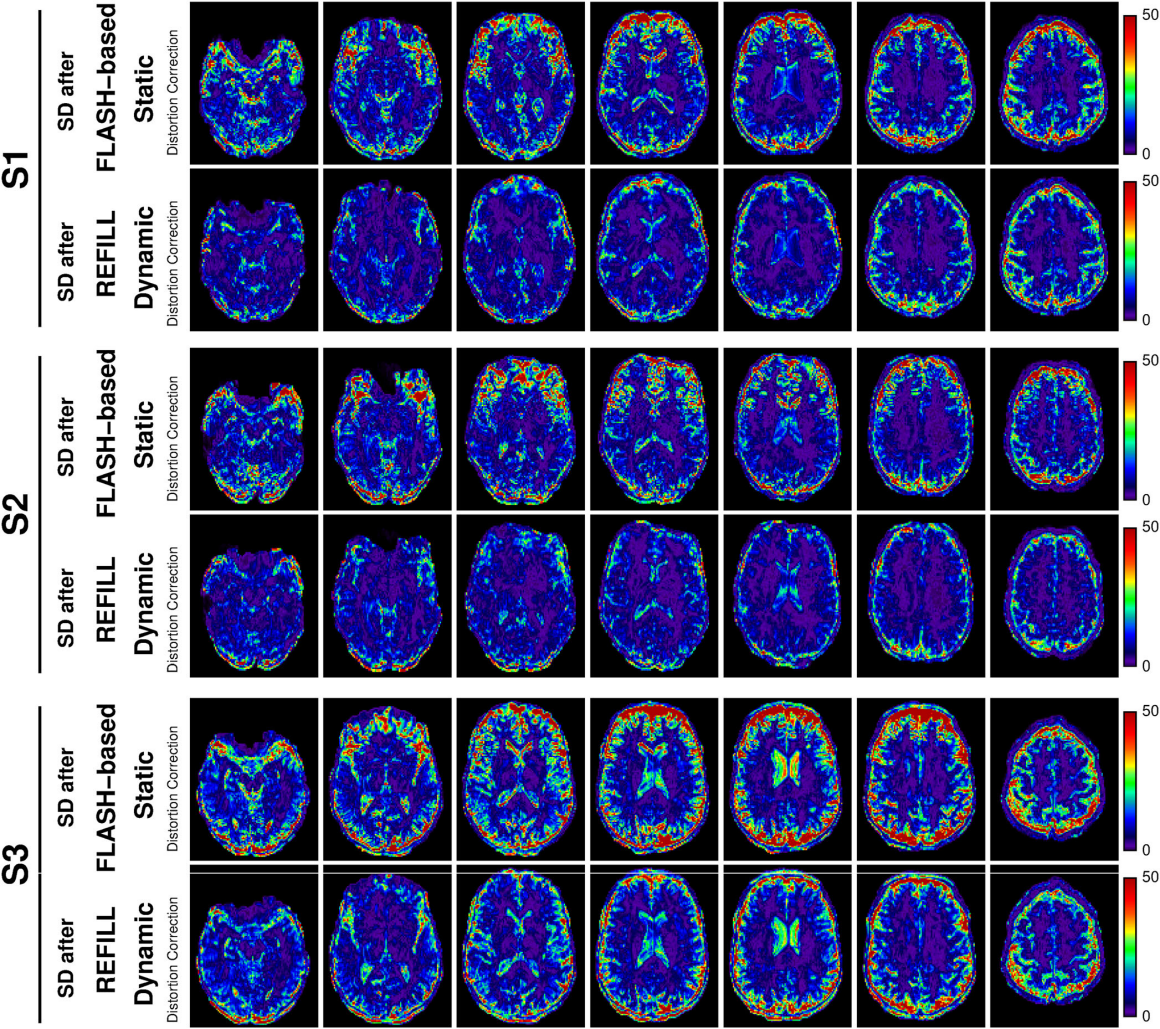
Comparison of time‐series standard deviation after static and DDC in three subjects who moved their hands towards their chin periodically to induce dynamic changes to the field at 7 T. Standard deviations were much smaller with the REFILL‐DDC correction. Movies of the uncorrected and corrected time series for S1, S2 and S3 are presented in Supporting Information in Movies S1–S3 respectively.

*Experiment 4: Accuracy and BOLD sensitivity of REFILL‐corrected time series in the presence of field changes*: In the case of a dynamically changing field (hand moving experiment), fluctuating distortions remained when the FLASH‐SDC was applied. These were apparent as high standard deviations of voxel values over time in Figure 6 (particularly at high contrast boundaries). Most dynamic distortions within the brain were removed in REFILL‐corrected time series, albeit with some residual fluctuations at the edge of the brain. The standard deviation of voxel values was much lower with REFILL (same figure). Residual motion, after (i) no distortion correction, (ii) FLASH‐SDC, and (iii) REFILL, each followed by motion correction, was much reduced in the REFILL + motion correction data (Figure S3). The differences between FLASH‐SDC and REFILL‐DDC data are best visualised in the movies in Supporting Information (Movies S1–S3 for subjects S1, S2 and S3, respectively).

**FIGURE 7:**
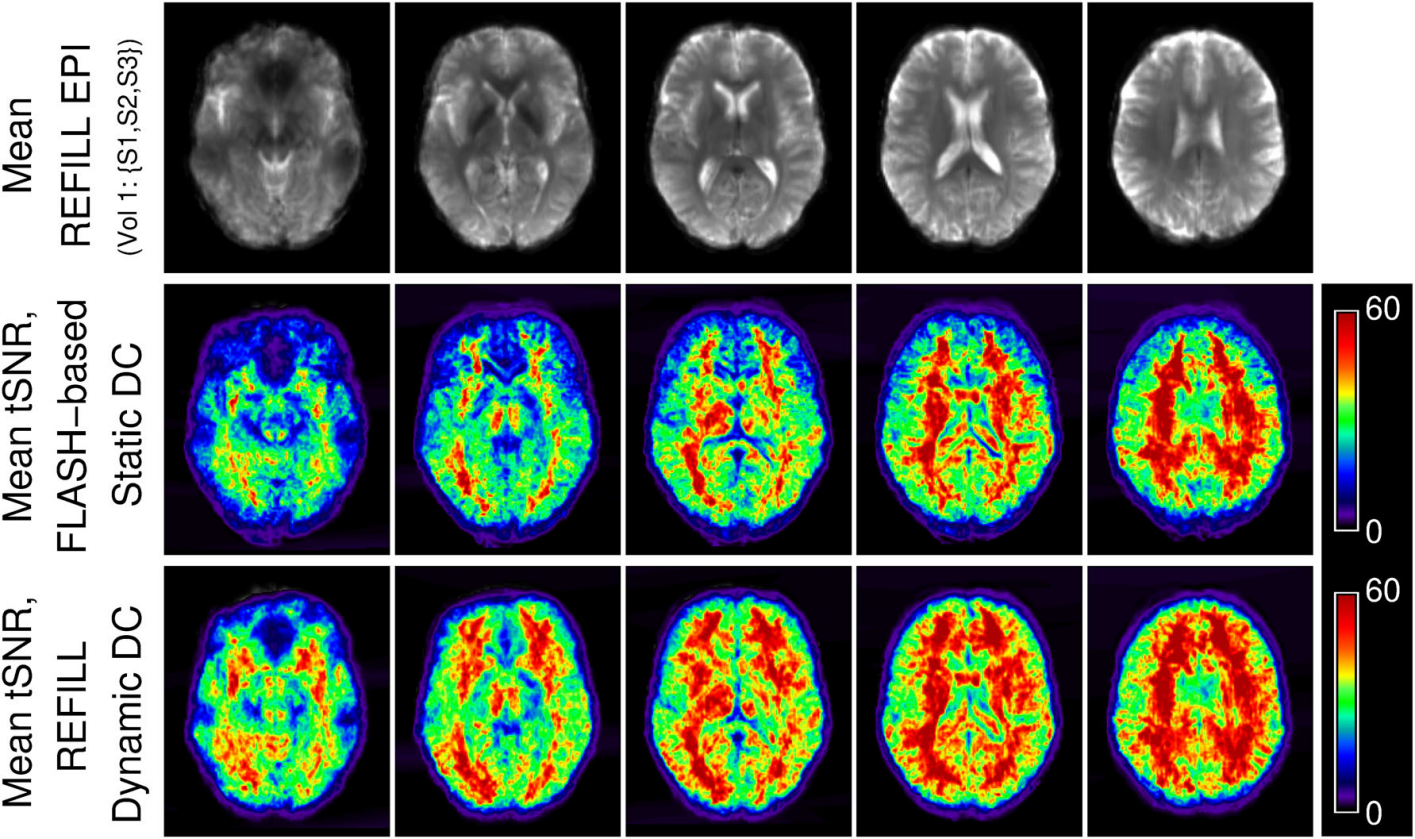
Comparison of mean tSNR, over 3 subjects, of FLASH‐SDC‐corrected and REFILL‐DDC‐corrected time‐series data during a task involving hand motion (tSNR maps co‐registered to MNI space). tSNR was higher over extended regions of the brain in the REFILL DDC‐corrected data.

Mean tSNR over subjects S1, S2 and S3 was higher by an average of 21.0% in grey matter in data corrected with REFILL‐DDC than FLASH‐SDC (Figure 7) (15.1% in white matter, 18.8% over the whole brain).

**FIGURE 8:**
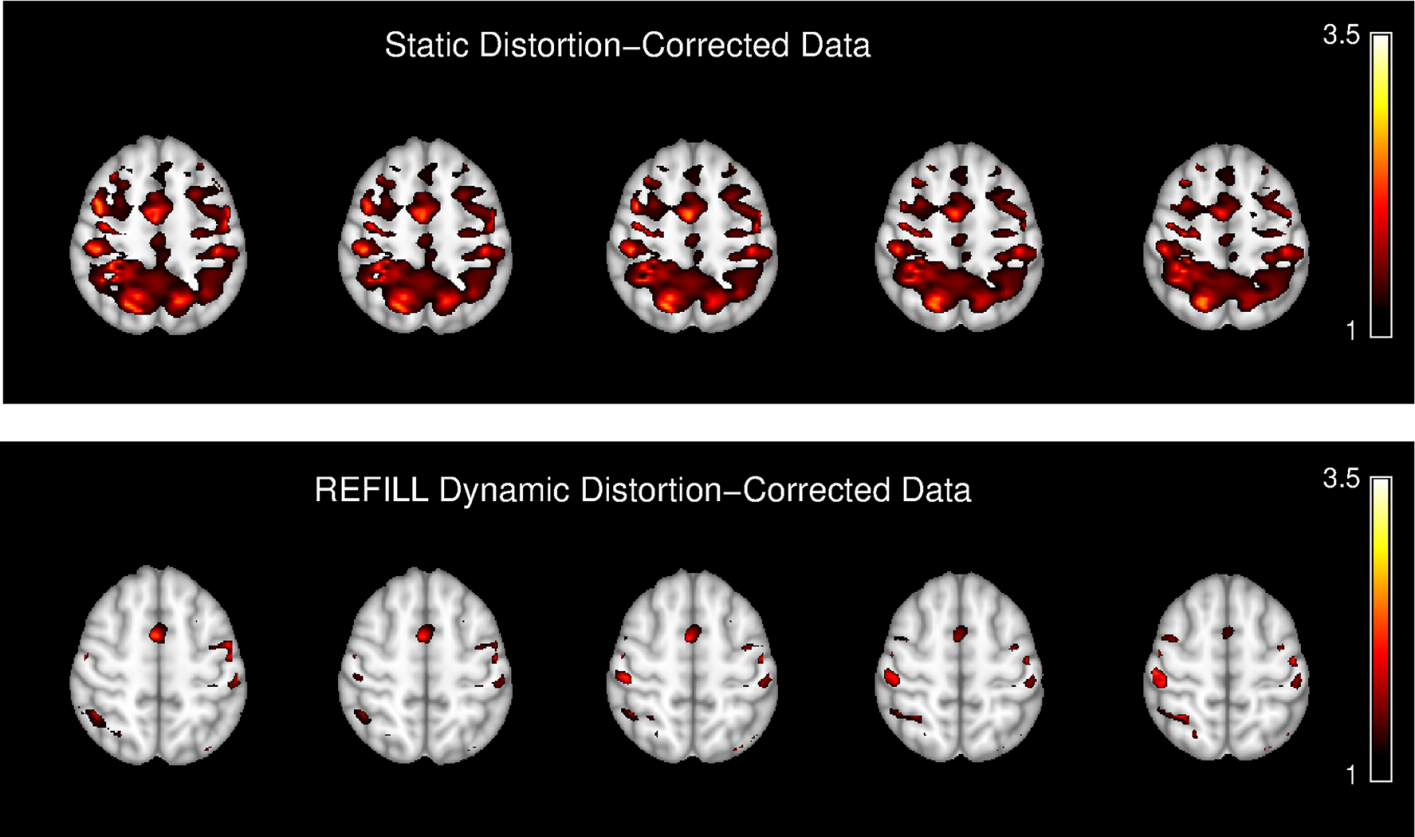
A comparison of activation maps between data corrected with a static distortion correction (top) and the proposed REFILL dynamic distortion method (bottom). fMRI results from data corrected with the static method show large regions of stimulus‐correlated distortion artefacts in addition to activation in primary motor and supplementary motor areas. REFILL‐corrected results show activation in known motor regions (bilateral hand, arm and supplementary motor area), with little or no residual motion‐related artefacts. Activation maps show results with *Z* > 1 with no cluster thresholding applied.

A functional analysis of the data acquired with the hand motion task shows that, if a static distortion correction is applied, extended areas of stimulus‐correlated distortions are present. These have *Z* values of similar value to those in motor regions activated by the task (Figure 8, top). In the REFILL, dynamic distortion‐corrected results, in contrast, only regions are visible which correspond to the task (primary hand and arm regions, supplementary motor area) (Figure 8, bottom).

## 5 DISCUSSION

We have presented an improved dynamic distortion correction method for fMRI which reduces the number of reference scans needed to calculate phase offsets to just one, of less than 10 s duration, and removes the need for spatial unwrapping in the calculation of phase offsets, making it possible to perform coil combination—the step which reduces the amount of data which needs to be stored—online. The bipolar reference scan and reversed‐readout EPI volume allow the identification and removal of non‐*B*_0_‐related contributions to the phase, so that the phase from each single‐echo EPI volume represents a wrapped, scaled field map. Online implementation of the key steps in REFILL allowed the method to be applied to conventional 2D EPI without the need for modifications to the sequence or access to the sequence source code. REFILL was insensitive to shim changes between the acquisition of the reference (coil sensitivity) scan and the EPI and accurately mapped the magnetic field as it changed over the course of the fMRI experiment due to motion, respiration and drift, leading to data with higher tSNR than with a static approach, and the virtual elimination of stimulus‐correlated distortion.

REFILL determines $\theta_{0}^{c}\;$from a single reference scan of less than 10 s, compared with two reference scans totalling nearly 90 s duration in the most recent single‐echo DDC method (Dymerska et al., 2018), and removes the need for unwrapping in the calculation of $\theta_{0}^{c}$, facilitating its implementation online. It also introduces a correction for non‐*B*_0_‐related phase gradient in the readout direction, $\varphi_{G\_\mathrm{EPI}}$, not present in the predecessor method, and efficient, phase‐based masking. The accuracy of $\theta_{0}^{c}\;$ estimates was shown to be independent of the prescan resolution and acceleration. The emergence of imaging artefacts in acquisitions with even lower resolution than that presented here was the limiting factor at 7 T.

The REFILL method introduces a correction for phase gradients in the readout direction. In this study, we found there to be a gradient of up to 3.2 × 10^−2^ rads/mm in EPI—equivalent to 55 Hz across a typical field of view for TE = 20 ms—and that this was strongly dependent on receiver bandwidth. Dymerska et al. (2018) did not report the presence of residual phase gradient in the readout gradient in single‐echo EPI. The discrepancy between those findings and ours is attributed to the use of difference EPI sequences and reconstructions between the two studies and indicates that this correction is, in general, necessary in order for DDC to be accurate. The phase gradient measured here was reduced by centring k‐space, suggesting that this source of phase variation could be reduced or removed with improved reconstruction, but also that this is not necessary for REFILL or the calculation of coil sensitivities according to Eckstein et al. (2018), as both methods include a readout gradient correction. The REFILL method uses a volume in which the readout direction is reversed. If integrated into the sequence this would take an additional TR of measurement time, but could also replace a dummy scan, in which case it would have no effect on the total acquisition time. There are other possibilities to correct the effect of $\varphi_{G\_\mathrm{EPI}}$, such as removing any global left–right gradient in field maps, but these would not be valid in the absence of symmetry in the object and shim, for example, with different gradient assignment or in the presence of pathologies.

In contrast to a FLASH‐based SDC, REFILL field maps were accurate when there was a change in shim, consistent with the finding of Dymerska et al. (2018) that estimated phase offsets are shim‐independent. The average FWHM of the difference between the FLASH‐based field map and the REFILL field map (both post shim‐change) was 11.7 ± 2.4 Hz, which corresponded to a discrepancy of 0.5 voxels, or 1 mm, in EPI. The sources of this residual difference were investigated. Respiration‐related changes in field between acquisitions were found to be much smaller than those related to small changes in head position, which together amounted to approximately 5 Hz. Other possible sources of difference between the two field maps relate to different PSF, poorly defined echo time in EPI, echo shifts in regions with strong gradients (Deichmann et al., 2002; Dymerska et al., 2019) and disparities in the effects of masking and interpolation.

We identified shortcomings in the TOPUP method: distortion maps contained streaks and features which do not accord with the anatomy, such as a shifted reproduction of the GM/WM border in the frontal cortex. In contrast to this finding, a number of previous studies have found PE‐reversed methods for SDC such as TOPUP to be more accurate than *B*_0_‐based methods. Most were carried out in the context of diffusion imaging, however, and used SE‐EPI (Graham et al., 2017; Holland et al., 2010). Some gradient‐echo‐based comparisons were performed at 3 T (Graham et al., 2017; Holland et al., 2010) or were carried out using spin‐echo reversed PE images (Yamamoto et al., 2021) but Schallmo et al. (2021) found that FLASH‐based PE‐reversed SDC slightly outperformed the *B*_0_ mapping‐based method at 7 T under essentially the same conditions as here. Given that the VSMs in Schallmo et al. (2021) seem to be patchy and noisy (Figure 5) it seems improbable that they represent more accurate measurements of the distortion field than VSMs from *B*_0_ mapping‐based methods. The more likely explanation for the better performance of TOPUP in that and other studies is that it provides estimates of distortions on the periphery of the brain and beyond which are more accurate than those from other methods.

Field maps need to be masked to remove unreliable values and interpolated to generate useful estimates of the field in regions affected by signal loss or in which there are errors, for example, due to flow or unwrapping errors. In the first paper proposing distortion correction with *B*_0_ field maps, Jezzard and Balaban used extensive processing of field maps including smoothing, polynomial fitting and constraining of estimates to fall off outside the object using dilated masking (Jezzard & Balaban, 1995). Hutton et al. (2002) also proposed sophisticated interpolation using 512 low frequency basis functions of the three‐dimensional discrete cosine set (DCS) of transformations. Common practice is reflected by the steps in the fsl_prepare_field map tool in FUGUE (Beisteiner et al., 2011; Triantafyllou et al., 2005; van der Zwaag et al., 2009); phase difference calculation, de‐spiking, de‐meaning, masking, unwrapping, a second de‐meaning step and a final despiking. It seems that the basis for the finding that reversed‐PE corrections such as TOPUP are better than *B*_0_ mapping methods is that the extrapolation of distortion estimates in some implementations is superior to even the elaborate processing described above.

Errors in the field estimations generated with TOPUP are interesting because of the overlap in the data needed for PE‐reversed SDC and REFILL: If both the readout polarity and the PE polarity are reversed in the reference EPI (which is the case, by default, on many systems), the data from the gradient‐reversed volume can be used either for TOPUP or REFILL. REFILL needs an additional short reference scan but provides a correction which is not only dynamic but also, on the basis of the limited comparison between the two methods here, more reliable.

Large dynamic distortions in EPI were well corrected with REFILL. There were residual distortions at the edge of the brain, arising both from spin history effects (with the changing field altering which slices of tissue are excited) and errors arising from the use of a single mask for the whole time series. In this study, a dedicated interpolation scheme had to be developed which was suited to 7 T, EPI‐based field maps. Signal inhomogeneity due to B_1_ inhomogeneity and EPI distortion make the use of magnitude‐based masking methods, such as BET, problematic. Instead, we generated masks for DDC field maps by thresholding an image quality map (Dymerska et al., 2021) which incorporated phase coherence; a method adopted by others recently in QSM (Bachrata, Trattnig, & Robinson, 2022; Hagberg et al., 2022; Stewart et al., 2022). These generated low‐noise field maps. Residual errors at the image boundary encountered with the quite extreme dynamic field fluctuations generated with the hand‐moving task at 7 T could be reduced by more sophisticated masking, including the generation of a mask for each image.

The REFILL method takes advantage of improved phase processing methods which allow phase offsets to be calculated without the need for spatial unwrapping (Eckstein et al., 2018) and channel‐combined EPI phase data to be unwrapped quickly and reliably (Dymerska et al., 2021). The avoidance of the need to unwrap single‐channel EPI data, as in a prior method (Dymerska et al., 2018), not only saves computational time, it allows dynamic field mapping with higher resolution (lower SNR) EPI. Preliminary tests with very high resolution EPI (0.8 mm isotropic), not presented here, show good results. Phase data generated in the REFILL process allow, in addition to dynamic distortion correction, data‐driven physiological noise correction (Bancelin et al., 2023), complex fMRI (Rowe, 2005), quantitative susceptibility mapping (QSM) (Bachrata, Bollmann, et al., 2022; Sun et al., 2017) and functional QSM (Balla et al., 2014).

In its current implementation, REFILL is performed as three separate scans; the fast coil sensitivity scan, the single readout‐reversed EPI volume and the EPI time series used for fMRI. This implementation allows it to be applied without needing to modify the EPI sequence, but this approach has two downsides. First, the acquisition geometry has to be set to be the same between the reference scan and fMRI acquisition, which introduces the possibility for user error. Second, on some systems the vendor implementation of pulse sequences requires the parallel imaging reference data to be acquired separately for each scan, which wastes some imaging time. Both of these drawbacks could be overcome by integrating the FLASH and REFILL volumes into the acquisition, although this would mean that the approach could not be used with other EPI sequences. The validity of the REFILL approach has been demonstrated for a ME‐FLASH reference scan and 2D EPI, the sequence used for vast majority of fMRI studies. We were able to apply the phase offsets calculated from the ME‐FLASH to the reconstruction of phase images from EPI, but acknowledge that it may not be trivial (or possible) to do this for sequences with different RF excitation pulses, trajectories (e.g. spiral), or sequences in which phase offsets are calculated using a different method, for example, as part of the reconstruction problem, as is the case in 3D EPI using CAIPIRINHA (Jin et al., 2021). In such cases it may be possible to calculate the difference between a field map generated from the reference scan and the first ‘functional’ volume and smooth and subtract that additional correction field from all subsequent time points. This may require the pre‐scan to be acquired with higher resolution and the assumption of temporal stability in this additional correction over the time series to be tested. Such a method would most closely resemble the TOAST approach (Hahn et al., 2009).

Dynamic distortion correction should be carried out before motion correction, making it the first step in fMRI pre‐processing. Our implementation is scripted in MATLAB and calls the compiled phase unwrapping programme ROMEO (Dymerska et al., 2021). As such, REFILL could be carried out as a stand‐alone process, prior to other pre‐processing, integrated into MATLAB‐based fMRI preprocessing (e.g. as part of SPM (Friston et al., 1995)) or (with rescripting in Python and Nipype (Gorgolewski et al., 2011)) integrated into a complete fMRI pre‐processing package such as fmriprep (Esteban et al., 2019).

A final limitation in this study relates to the assessment of the accuracy of the field maps acquired with REFILL. These were compared with FLASH‐based field maps in vivo, but those do not offer a perfect measurement of the field. Our assessment of the contribution of $\varphi_{G}$ to the phase and the effect of shifting the echo in reconstruction reinforce the point that FLASH‐based field estimates are sensitive to a number of imaging parameters (beyond the echo time), as has been shown by others (Varadarajan et al., 2021). Also, the small residual discrepancies between field estimates obtained with REFILL and the FLASH‐based approach were observed to be located primarily in white matter, which is known to exhibit non‐linear phase evolution (Wharton & Bowtell, 2012), making a comparison of field estimates derived from measurements with different echo times somewhat problematic. Furthermore, while the in vivo condition ensured that field variations were realistic, there were inevitably minor residual discrepancies between the field in the two acquisitions due to respiration and motion, even in experienced and well‐instructed subjects. Nevertheless, where static field maps (from a pre‐shim‐change FLASH‐based field map or TOPUP) were wrong by up to 100 Hz, the difference between REFILL and the field measured in the changed shim condition was only a few Hz, confirming that the sum of these confounding effects is below the level which would affect distortion correction.

In summary, the REFILL single‐echo DDC approach introduces a correction for a phase gradient in the readout direction in EPI which we show to be necessary to achieve an accurate distortion correction. In comparison to the original method, the number of sensitivity scans is reduced from two to one, and the duration of the sensitivity scan has been reduced approximately by a factor of 10, to around 5 s. The adoption of a recently developed method of phase offset correction removes the need for phase unwrapping in the online calculations, while improved phase unwrapping and masking has made accurate dynamic field mapping practicable. REFILL requires no additional hardware, just a few seconds of additional scan time and provides an effective distortion correction even with large field changes at ultra‐high field, making it feasible to apply dynamic distortion correction routinely in fMRI.

## Supporting information


**Figure S1.** Dependence of REFILL fieldmap values on the use of fat saturation, resolution and acceleration in the multi‐echo GE reference scan. Values are the differences between REFILL fieldmaps calculated using reference scans with different image parameters and a gradient‐echo fieldmap. REFILL fieldmaps generated using phase offsets derived from an acquisition with no fat saturation (TA = 25 s, red line in top plot) agree well with those acquired with fat saturation (TA = 53 s, black line in top plot). There was also no substantial change in fieldmaps when a highly accelerated (GRAPPA 4) low resolution reference scan with an acquisition time of 3 s was used (the green line in the lower histogram) compared to a reference scan with GRAPPA 2, fat saturation, the same resolution as the EPI and an acquisition time of 53 s (black line in the same histogram). Example images from one channel (channel 2) for the two acquisitions illustrate the fact that the phase offset is spatially smooth, and well captured by the TA = 3 s acquisition
**Figure S2.** Changes in *B*_0_ related to respiration and small changes in head position. FLASH‐FMs acquired at inhalation and exhalation (left) differ by approximately 1 Hz (FWHM of histogram). A small change in head position (2.43 mm root‐mean‐squared voxel shift), in contrast, leads to differences of circa 4 Hz FWHM (right).
**Figure S3.** Distortion correction and motion correction. The hand‐to‐chin task described in the main text led to the mean image displacements identified (by FSL's mcflirt) in the left column. A second pass of motion correction showed that residual motion effects were much smaller in the case of REFILL (red boxes), as rigid‐body motion correction could not remove dynamic distortion in raw and SDC‐corrected images

Video S1.

Video S2.

Video S3.

## Data Availability

Data and results are publicly available at https://doi.org/10.7910/DVN/ZWNZXM. The on‐console reconstruction is available as part of the ASPIRE package for Siemens NUMARIS software versions VB17, VE11C and VE12U via C2P agreement (contact simon.robinson@meduniwien.ac.at) and the REFILL method for data reconstructed using ASPIRE is available on github at https://github.com/simon-mri/REFILL-Dynamic-Distortion-Correction. An offline reconstruction for separate‐channel data is also available https://github.com/korbinian90/ASPIRE. These results have previously been reported in short and preliminary form (Robinson et al., 2021, 2022).
