# Supplementary material for: Improved dynamic distortion correction for fMRI using single‐echo EPI and a readout‐reversed first image (REFILL)
Source: Hum Brain Mapp. 2023 Aug 7;44(15):5095–112. doi: 10.1002/hbm.26440 (PMC10502646; doi:10.1002/hbm.26440)
Supplement: Supplementary file 1 — Figure S1. Dependence of REFILL fieldmap values on the use of fat saturation, resolution and acceleration in the multi‐echo GE reference scan. Values are the differences between REFILL fieldmaps calculated using reference scans with different image parameters and a gradient‐echo fieldmap. REFILL fieldmaps generated using phase offsets derived from an acquisition with no fat saturation (TA = 25 s, red line in top plot) agree well with those acquired with fat saturation (TA = 53 s, black line in top plot). There was also no substantial change in fieldmaps when a highly accelerated (GRAPPA 4) low resolution reference scan with an acquisition time of 3 s was used (the green line in the lower histogram) compared to a reference scan with GRAPPA 2, fat saturation, the same resolution as the EPI and an acquisition time of 53 s (black line in the same histogram). Example images from one channel (channel 2) for the two acquisitions illustrate the fact that the phase offset is spatially smooth, and well captured by the TA = 3 s acquisition Figure S2. Changes in B 0 related to respiration and small changes in head position. FLASH‐FMs acquired at inhalation and exhalation (left) differ by approximately 1 Hz (FWHM of histogram). A small change in head position (2.43 mm root‐mean‐squared voxel shift), in contrast, leads to differences of circa 4 Hz FWHM (right). Figure S3. Distortion correction and motion correction. The hand‐to‐chin task described in the main text led to the mean image displacements identified (by FSL's mcflirt) in the left column. A second pass of motion correction showed that residual motion effects were much smaller in the case of REFILL (red boxes), as rigid‐body motion correction could not remove dynamic distortion in raw and SDC‐corrected images [file HBM-44-5095-s003.docx]

| 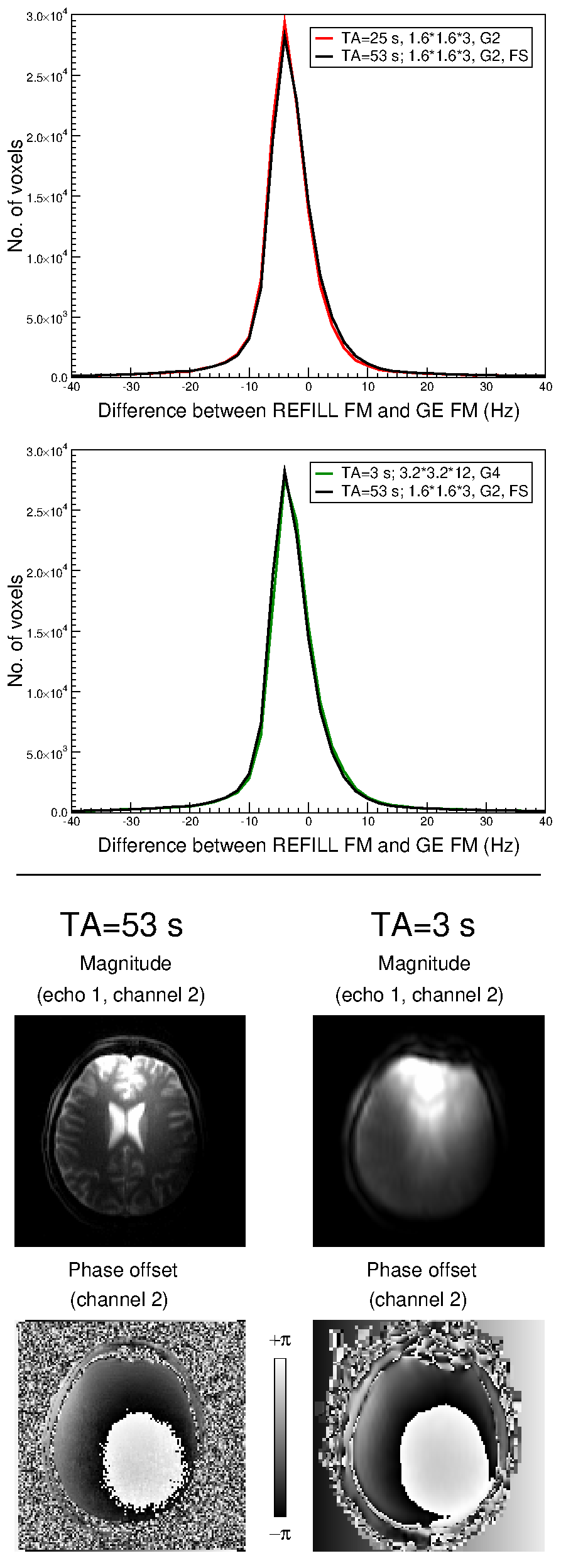 |
| --- |
| ***sFigure 1****: Dependence of REFILL fieldmap values on the use of fat saturation, resolution and acceleration in the multi-echo GE reference scan. Values are the differences between REFILL fieldmaps calculated using reference scans with different image parameters and a gradient-echo fieldmap. REFILL fieldmaps generated using phase offsets derived from an acquisition with no fat saturation (TA=25s, red line in top plot) agree well with those acquired with fat saturation (TA=53s, black line in top plot). There was also no substantial change in fieldmaps when a highly accelerated (GRAPPA 4) low resolution reference scan with an acquisition time of 3 seconds was used (the green line in the lower histogram) compared to a reference scan with GRAPPA 2, fat saturation, the same resolution as the EPI and an acquisition time of 53 seconds (black line in the same histogram). Example images from one channel (channel 2) for the two acquisitions illustrate the fact that the phase offset is spatially smooth, and well captured by the TA=3 s acquisition.* |

| 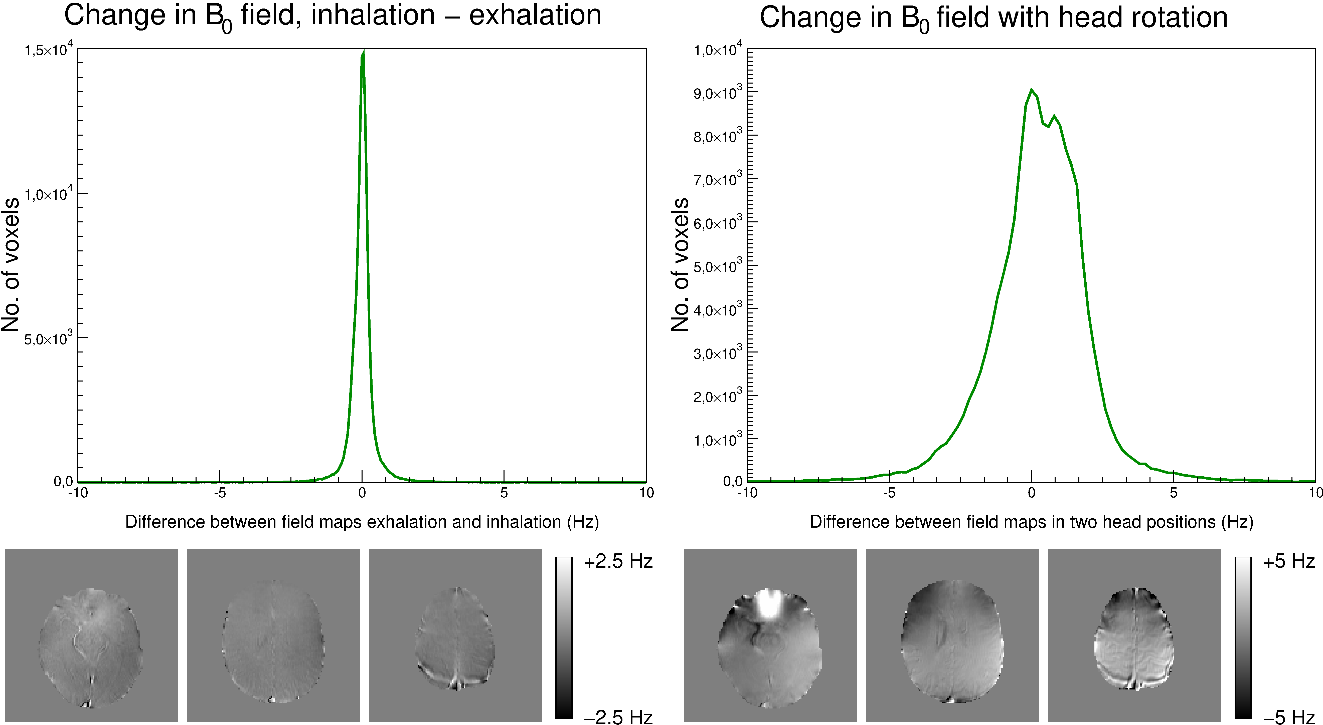 |
| --- |
| ***sFigure 2****: Changes in B_0_ related to respiration and small changes in head position. FLASH-FMs acquired at inhalation and exhalation (left) differ by approximately 1 Hz (FWHM of histogram). A small change in head position (2.43 mm root-mean-squared voxel shift), in contrast, leads to differences of circa 4 Hz FWHM (right).* |

| 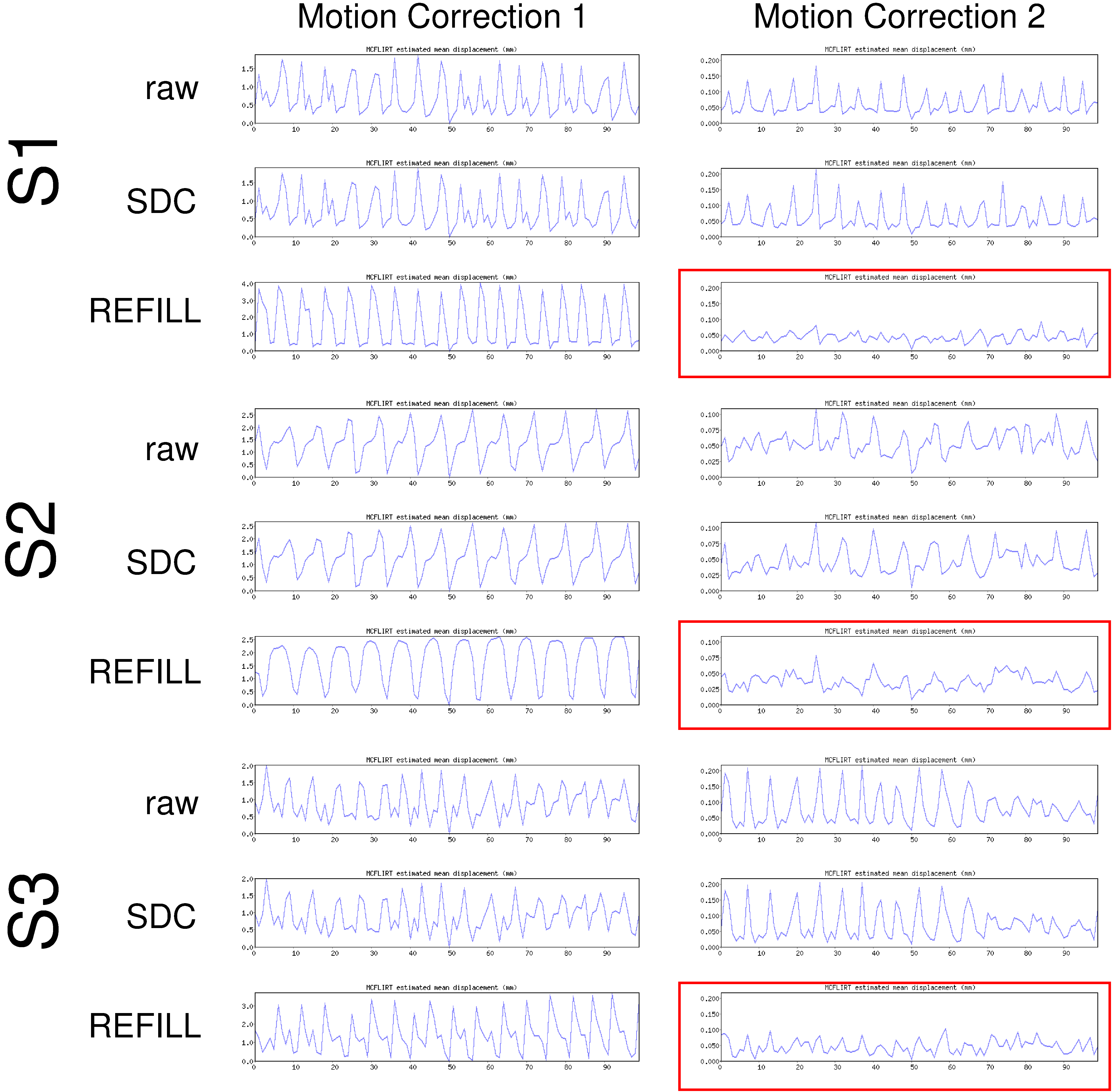 |
| --- |
| ***sFigure 3****: Distortion correction and motion correction. The hand-to-chin task described in the main text led to the mean image displacements identified (by FSL’s mcflirt) in the left column. A second pass of motion correction showed that residual motion effects were much smaller in the case of REFILL (red boxes), as rigid-body motion correction could not remove dynamic distortion in raw and SDC-corrected images.* |
